# Cognitive impairment of patients with chronic renal disease on hemodialysis and its relationship with sociodemographic and clinical characteristics

**DOI:** 10.1590/1980-57642016dn11-030003

**Published:** 2017

**Authors:** Gabriela Dutra Gesualdo, Juliana Gomes Duarte, Marisa Silvana Zazzetta, Luciana Kusumota, Karina Gramani Say, Sofia Cristina Iost Pavarini, Fabiana de Souza Orlandi

**Affiliations:** 1 Doutoranda em Ciências da Saúde pelo Programa de Pós Graduação em Enfermagem Fundamental, Escola de Enfermagem de Ribeirão Preto, Universidade de São Paulo, Ribeirão Preto SP, Brasil.; 2 Discente do Curso de Graduação em Gerontologia, Departamento de Gerontologia, Universidade Federal de São Carlos, São Carlos SP, Brasil.; 3 Docente do Curso de Graduação em Gerontologia, Departamento de Gerontologia, Universidade Federal de São Carlos, São Carlos SP, Brasil.; 4 Docente da Escola de Enfermagem de Ribeirão Preto, Universidade de São Paulo, Ribeirão Preto SP, Brasil.

**Keywords:** aged, renal dialysis, renal insufficiency chronic, cognition, idoso, diálise renal, insuficiência renal crônica, cognição

## Abstract

Cognitive impairment and dementia commonly occur in individuals with chronic kidney disease, especially in advanced stages, but are still poorly diagnosed.

**OBJECTIVE:**

To evaluate the cognitive ability of patients with chronic kidney disease on hemodialysis and its relationship with sociodemographic and clinical characteristics.

**METHODS:**

A cross-sectional study was carried out in a Renal Replacement Therapy Unit in the interior of the State of São Paulo involving 99 patients. The data were collected through an individual interview, using the Sociodemographic and Clinical Characterization questionnaires and the Addenbrooke's Cognitive Examination – Revised (ACE-R) questionnaire.

**RESULTS:**

Participants were predominantly male, with a mean age of 54.68 years. The mean ACE-R score was 64.26 points, and 76.76% of patients had lower-than-expected scores, suggesting the presence of cognitive impairment. A moderate, negative correlation was found between total score on the ACE-R and age (r= –0.38, p≤0.001), a moderate positive correlation with years of education (r=0.52, p≤0.001), and a weak positive correlation of total score with hemodialysis time (r=0.26, p≤0.001).

**CONCLUSION:**

A relationship was found between cognitive ability and age, years of education and hemodialysis time, suggesting that individuals who were older, had less education and longer hemodialysis time presented greater cognitive impairment.

## RESUMO

O comprometimento cognitivo e demência ocorrem comumente em indivíduos com doença renal crônica (DRC), principalmente em fase avançada, mas ainda são pouco diagnosticados.

**OBJETIVO:**

Avaliar a capacidade cognitiva de pacientes com doença renal crônica em hemodiálise e a sua relação com as características sociodemográficas e clínicas.

**MÉTODOS:**

Trata-se de um estudo correlacional, de corte transversal, realizado em uma Unidade de Terapia Renal Substitutiva do interior do Estado de São Paulo, com 99 pacientes. Os dados foram coletados por entrevista individual, utilizando-se os questionários de Caracterização Sociodemográfica e Clínica e o *Addenbrooke's Cognitive Examination – Revised* (ACE-R).

**RESULTADOS:**

A maioria dos participantes era do gênero masculino, com idade média de 54,68 anos. A pontuação média do ACE-R foi de 64,26 pontos, sendo que 76,76% (n=76) apresentaram pontuação abaixo do esperando, sugerindo a presença de comprometimento cognitivo e verificou-se correlação negativa, de moderada magnitude, entre a pontuação total do ACE-R e a idade (r= –0,38; p-valor≤0,001), correlação positiva, de moderada magnitude, com os anos de escolaridade (r=0,52; p-valor≤0,001) e correlação positiva de fraca magnitude com o tempo de hemodiálise (r=0,26; p-valor≤0,001).

**CONCLUSÃO:**

Houve relação entre a capacidade cognitiva, a idade, os anos de escolaridade e tempo de hemodiálise sugerindo que as pessoas mais idosas, com menor escolaridade e maior tempo de hemodiálise apresentaram mais comprometimento cognitivo.

## INTRODUCTION

Cognitive impairment and dementia commonly occur in individuals with chronic kidney disease (CKD), especially in advanced stages, but are still poorly diagnosed.^1^ Dementia is a state of persistent and progressive cognitive dysfunction characterized by impairment in memory and in at least one other aspect, or domain, of cognitive function, such as language, orientation, reasoning, attention, or executive functioning - the cognitive skill necessary for planning and sequencing tasks.^2^ The term cognitive impairment is used to describe a deficit beyond that associated with normal aging, but not characterized as dementia.

However, individuals at any stage of the disease are susceptible to cognitive dysfunction, associated with increased risk of death, poor adherence to recommended treatments, increased progression of cerebrovascular disease and longer hospitalizations.^3^^-^ 5 CKD maybe strongly associated with the incidence of dementia, especially Vascular Dementia,^6^^,^ 7 relative to the general population. The prevalence of cognitive impairment is higher in individuals with renal failure, especially those on dialysis.^8^ Mild cognitive impairment differs from the dementia syndrome and, unlike dementia, has no impact on individual daily activities.

The etiology of cognitive impairment is multifactorial, but the chronic and debilitating nature of CKD, coupled with an exhaustive routine of treatments, may be responsible for this change.^9^ The impairment in cognitive function is also attributed to the effect of uremic toxins.^10^

The risk of cognitive impairment and dementia in patients with CKD is due to the high prevalence of ischemic, symptomatic or asymptomatic cerebrovascular injuries.^3^ This vascular mechanism may explain the association between risk factors affecting both the brain and the kidneys and their potential exacerbation in renal disease.^11^

Oxidative stress, immune inflammatory processes, anemia, hyperhomocysteinemia, and vitamin B12 deficiency may be involved with this decline in neurocognitive performance. Patients who present CKD and undergo hemodialysis have more prothrombotic factors, such as endothelial dysfunction, abnormal vascular reactivity, atherosclerosis, and cardiovascular events.^12^^-^ 17

In a recent study comparing the cognitive performance of dialysis patients with that of the general population found that respondents on dialysis had worse performance in tasks evaluating executive function, associated with vascular disease and risk factors.^18^

Individuals undergoing hemodialysis had worse performance on tests evaluating logical reasoning, verbal learning, motor ability, verbal fluency and visuospatial ability. Dialysis processes directly contribute to cognitive decline by inducing cerebral ischemia. Acute reduction in intravascular volume and fluid changes that occur during sessions can cause edema and reduce cerebral perfusion.^19^

Despite evidence that early hemodialysis improves cognitive abilities in patients with CKD, a large proportion of hemodialysis patients present moderate to severe cognitive impairment, even if poorly diagnosed. In a review study, no difference in cognitive dysfunction was found among patients with CKD treated using different dialysis modalities.^20^

Cognitive assessment in individuals with CKD is of the utmost importance. Due to the complexity of this condition, patients have to process and understand significant amounts of information in order to properly comply with the treatment. Early diagnosis and intervention help contain or mitigate the progress of cognitive impairment.^20^ Thus, the objective of the present study was to evaluate the cognitive ability of patients with chronic renal disease on hemodialysis, and its relationship with sociodemographic and clinical characteristics.

## METHODS

### Participants.

A cross-sectional study with a quantitative approach was conducted in a Renal Replacement Therapy Unit situated in the interior of the State of São Paulo, Brazil. During the data collection period, 165 patients benefited from the Unified Health System and agreements, covering the cities of the municipality of São Carlos. The sample was designed for convenience, with a total of 99 participants, who met the following eligibility criteria: age 20 years or older, medical diagnosis of terminal stage CKD (category 5) and hemodialysis treatment for at least 6 months. Participants were excluded from the sample if: [A] younger than 20 years old; [B] treated by peritoneal dialysis; [C] on hemodialysis for less than 6 months; [D] patients exhibiting sequelae of stroke; [E] patients with severe visual or hearing impairment. After the invitation to participants, the objective of the present study was explained and any queries answered. Having agreed to participate in the study, all respondents signed the informed consent form. All ethical guidelines were respected and the project was approved by the Research Ethics Committee of the Federal University of São Carlos under permit nº. 799.212/2014.

### Measures.

A multi-dimensional questionnaire was applied to assess sociodemographic and clinical aspects. The following sociodemographic variables were analyzed: age, gender and educational level. Clinical aspects were analyzed based on the following variables: hemodialysis time, albumin, hematocrit, urea levels and health status.

### Addenbrooke's Cognitive Examination – Revised (ACE-R) questionnaire.

This was developed in the United Kingdom in 2000 and reviewed by Mioshi et al. in 2006, to facilitate its application and include new language issues. The ACE-R was adapted and validated for use in Brazil by Carvalho and Caramelli in 2007.^21^

The questionnaire contains five subscores, each representing a cognitive domain. The general ACE-R score ranges from 0 to 100 points, distributed across five domains: orientation/attention (18 points), memory (26 points), verbal fluency (14 points), language (26 points) points and visuospatial abilities (16 points), all of which are evaluated individually. Cut-off scores indicating cognitive impairment have been established for the overall battery, as well as for each domain and the Mini-Mental State Examination (MMSE) included in the ACE-R. These cut-off scores are: <78 points for total, 17 points for orientation/attention, <15 points for memory, <8 points for verbal fluency, <22 points for language, <13 for visuospatial ability, and <25 for the MMSE included in the ACE-R.^21^

### Statistical analysis.

The data were keyed into a spreadsheet in Excel for Windows 7 and the descriptive analysis was carried out using measures of position (mean, median, minimum and maximum) and dispersion (standard deviation) with the software SPSS (Statistical Package for the Social Sciences) version 22.0. The Shapiro-Wilk normality test was also performed, revealing non-normal distribution of data. Spearman's Correlation Coefficient was calculated between ACE-R (total and domain) scores and the sociodemographic and clinical variables. In this study, the magnitudes of the correlations were classified according to Levin and Fox (2014): weak (<0.3); moderate (0.3 to 0.59), strong (0.6 to 0.9) and perfect (1.0). The level of significance adopted for the statistical tests was 5%, p-value≤0.05.

## RESULTS

The majority of patients with CKD were male (67.67%, n=67), with a mean age of 54.68 (±15.19) years, ranging from 22 to 85 years. Mean years of education was 7.37 (±4.18) years, whereas mean hemodialysis time was 54.98 (±54.06) months. Regarding health status, the majority (56.56%) of participants described their health as excellent, very good or good (Table 1).

**Table 1:** Distribution of patients with chronic renal disease according to sociodemographic and clinical characteristics.

Variable	Mean (±sd*)	Median	Observed variation	Distribution into categories	n	%
Gender	–	–	–	Male	67	67.67
				Female	32	32.33
Age (years)	54.68 (±15.19)	57.00	22-85	22-31	6	6.06
				32-41	15	15.15
				42-51	19	19.19
				52-61	23	23.23
				62-71	19	19.19
				72-81	15	15.15
				82-91	2	2.03
Education (years)	7.37 (±4.18)	8.00	0-20	0-5	41	41.41
				6-10	30	30.30
				11-15	24	24.24
				16-20	4	4.05
Hemodialysis time (months)	54.98 (±54.06)	41.00	6-264	6 Ⱶ 18	25	25.25
				18 Ⱶ 30	17	17.17
				30 Ⱶ 42	5	5.06
				42 Ⱶ 54	11	11.11
				54 Ⱶ 66	12	12.12
				66 ├┤ 264	29	29.29
Health status	–	–	–	Excellent/Very Good/Good	56	56.56
				Regular/Poor	40	40.40
				Very poor	3	3.04

* standard deviation.

Regarding the cognitive performance of the CKD patients, mean score on the ACE-R was 64.26 (±18.80) points. The domain with the highest mean score was "Language" 19.63 (±5.92) while the domains exhibiting the greatest deficit compared to the cut-off scores were "Orientation/Attention" with a mean of 14.33 (±3.29) and "Visuospatial Ability" with a mean of 8.55 (±3.70) (Table 2). Regarding the cognitive evaluation, 76.76% (n=76) had lower-than-expected scores, suggesting the presence of some degree of deficit.

**Table 2:** Descriptive analysis of domains evaluated by ACE-R in patients with chronic kidney disease.

Domains	Maximum score	Cut-off score	Mean (±sd*)	Median	Observed variation
Orientation / Attention	18	<17	14.33 (±3.29)	15	3-18
Memory	26	<15	14.37 (±5.87)	16	0-26
Verbal Fluency	14	<8	7.41 (±3.99)	8	0-14
Language	26	<22	19.63 (±5.92)	22	0-26
Visuospatial Ability	16	<13	8.55 (±3.70)	8	0-16
Total Score	100	<78	64.26 (±18.80)	68	6-97

* standard deviation.

Concerning the relationship between cognition and sociodemographic and clinical variables, there was a moderate correlation between total ACE-R score and both age (r= –0.38, p≤0.001) and education (r=0.52, p≤0.001). The "Orientation/Attention" domain correlated positively with both years of education (r=0.40, p≤0.001) and hemodialysis time (r=0.26, p≤0.001). For the memory domain, there was a weak negative correlation with age (r= –0.20, p=0.04) and a positive correlation with both years of education (r=0.42, p≤0.001) and hemodialysis time (r=0.21, p=0.03) (Table 3)

**Table 3:** Spearman's correlation coefficient between ACE-R and its domains and sociodemographic and clinical variables of patients with chronic kidney disease.

		Age	Years of education	Hemodialysis time
ACE-R	Spearman correlation	–0.38	0.52	0.18
	P	≤0.001	≤0.001	0,07
Orientation and Attention	Spearman correlation	–0.13	0.40	0.26
	P	0.17	≤0.001	≤0.001
Memory	Spearman correlation	–0.20	0.42	0.21
	P	0.04	≤0.001	0.03
Verbal Fluency	Spearman correlation	–0.07	0.31	0.04
	P	0.48	≤0.001	0.66
Language	Spearman correlation	–0.43	0.48	0.13
	P	≤0.001	≤0.001	0.19
Visuospatial Ability	Spearman correlation	–0.40	0.40	0.09
	P	≤0.001	≤0.001	0.34

## DISCUSSION

The profile of the patients studied was similar to those found in similar studies involving participants with CKD on hemodialysis in Brazil and other countries.^22^^,^ 23 Previous studies suggest that the greater the severity of CKD, the greater the progression of cognitive decline.^24^ In a study of 1315 hemodialysis patients in the city of Singapore, decreasing estimated GFR and the presence of CKD in patients over 55 years of age were associated with greater overall cognitive decline at four years of follow-up.^25^ Another investigation involving 7839 participants with CKD on hemodialysis failed to demonstrate that increased risk of cognitive impairment or dementia was associated with low estimated GFR.^11^

In a study investigating the association between level of physical activity and cognitive function on the MMSE of 102 patients with CKD on hemodialysis at the Clinicas Hospital of the Medical School of Botucatu, 44.1% of participants presented mild to severe cognitive deficit and higher level of physical activity was associated with better cognitive function,^26^ similar findings to those of the present study.

The hemodialysis process and/or the intense catabolism characteristic of CKD can contribute to and even accelerate brain aging, since compared to healthy individuals, hemodialysis patients had worse performance on cognitive tests, despite having a mean age less than or equal to that of the other participants.^9^^,^ 10 Similarly, an investigation that evaluated the cognitive ability of 75 chronic renal patients on hemodialysis using the MMSE found a mean score of 24.16 (±4.49) points, with a minimum of seven and maximum of 30 points. In addition, the domain with the lowest score was "Attention" and "Calculation",^20^ data corroborated by the present study.

In a recent study of 314 patients on dialysis at a Boston Dialysis Clinic, compared to the general population, patients had worse performance on tasks assessing executive function, associated with vascular disease and vascular risk factors.^18^ In the present study, hemodialysis subjects showed worse performance on tests evaluating logical reasoning, verbal learning, motor ability, verbal fluency and visuospatial memory.

In an investigation of cognitive function, depression and quality of life of patients at different stages of CKD, in which 119 patients on hemodialysis, peritoneal dialysis and conservative treatment were evaluated using the MMSE as a tool for cognitive screening, the hemodialysis patients had worse performance on the cognitive evaluation tests when compared to the peritoneal dialysis and conservative treatment groups.^9^

A study evaluating the cognitive ability of individuals with CKD on hemodialysis using the MMSE as well as the relationship with the sociodemographic and clinical characteristics of these individuals, found a mean score of 24.16 points on the MMSE and no difference for causes of CKD.^20^ In the study, MMSE scores correlated positively with years of education and income per capita and inversely with age.

CKD has been strongly correlated with vascular dementia in particular,^7^ relative to the general population, and the prevalence of cognitive impairment is higher in individuals with renal failure and even more significant in patients on dialysis. In qualitative terms, mild cognitive impairment is similar to dementia, although the impact of the condition on the performance of activities of daily living is not as significant as that caused by dementia.^27^

Dementia appears to complicate the management of patients with ESRD and has been correlated with poorer prognosis. Patients with dementia present deficits in at least two of the following cognitive functional areas: memory, executive function, attention, visuospatial abilities, speed of information processing, and language.^27^ Although evidence suggests that hemodialysis improves cognition in patients with CKD,^5^ a significant, yet underestimated, portion of individuals on hemodialysis has moderate to severe cognitive impairment.^27^

Based on our results, it was concluded that there was a relationship between cognitive ability and age, years of education and hemodialysis time, where older individuals with less education and more time on hemodialysis had lower cognitive performance.

The identification of patients with cognitive disorders is important to improve quality of life and reduce the morbidity associated with this condition. The ideal timing for the assessment of cognitive function and the instruments to be used for diagnostic screening depend on the clinical situation of the patient.

Finally, further research is needed to devise and evaluate preventive strategies for these factors in chronic kidney disease patients in order to delay or avert cognitive deficit in these patients.
